# Investigating the Relationship between Socially-Assigned Ethnicity, Racial Discrimination and Health Advantage in New Zealand

**DOI:** 10.1371/journal.pone.0084039

**Published:** 2013-12-31

**Authors:** Donna M. Cormack, Ricci B. Harris, James Stanley

**Affiliations:** 1 Department of Public Health, University of Otago, Wellington, New Zealand; 2 Dean's Department, University of Otago, Wellington, New Zealand; Rollins School of Public Health, United States of America

## Abstract

**Background:**

While evidence of the contribution of racial discrimination to ethnic health disparities has increased significantly, there has been less research examining relationships between ascribed racial/ethnic categories and health. It has been hypothesized that in racially-stratified societies being assigned as belonging to the dominant racial/ethnic group may be associated with health advantage. This study aimed to investigate associations between socially-assigned ethnicity, self-identified ethnicity, and health, and to consider the role of self-reported experience of racial discrimination in any relationships between socially-assigned ethnicity and health.

**Methods:**

The study used data from the 2006/07 New Zealand Health Survey (n = 12,488), a nationally representative cross-sectional survey of adults 15 years and over. Racial discrimination was measured as reported individual-level experiences across five domains. Health outcome measures examined were self-reported general health and psychological distress.

**Results:**

The study identified varying levels of agreement between participants' self-identified and socially-assigned ethnicities. Individuals who reported both self-identifying and being socially-assigned as always belonging to the dominant European grouping tended to have more socioeconomic advantage and experience less racial discrimination. This group also had the highest odds of reporting optimal self-rated health and lower mean levels of psychological distress. These differences were attenuated in models adjusting for socioeconomic measures and individual-level racial discrimination.

**Conclusions:**

The results suggest health advantage accrues to individuals who self-identify and are socially-assigned as belonging to the dominant European ethnic grouping in New Zealand, operating in part through socioeconomic advantage and lower exposure to individual-level racial discrimination. This is consistent with the broader evidence of the negative impacts of racism on health and ethnic inequalities that result from the inequitable distribution of health determinants, the harm and chronic stress linked to experiences of racial discrimination, and via the processes and consequences of racialization at a societal level.

## Introduction

As in many other countries with histories of colonization, New Zealand has entrenched inequities in health between ethnic groups, with persistent disparities in morbidity and mortality rates, life expectancy, and access to and experiences of healthcare [1]. Measuring and monitoring health disparities and other social outcomes between ethnic groups often uses administrative or routinely-collected data. In New Zealand, official statistical approaches to ethnicity are formally based on the concept that individuals are able to self-identify with one or more ethnic groups [2]. This has been a change over time from historical approaches based on ancestry or ‘blood quantum’ to a contemporary understanding of ethnicity as a measure of self-identified cultural affiliation [2]. Accompanying this has been a shift in terminology away from ‘race’ to use of the term ‘ethnicity’ in official statistics in New Zealand, although the term ‘race’ continues to be used interchangeably with ‘ethnicity’ in many social contexts, as is the case internationally [3].

While self-identification is now generally accepted as a central tenet of official approaches to ethnicity data collection, in everyday social interactions an individual's ‘race’ or ethnicity is also socially-assigned. Socially-assigned race/ethnicity relates to an understanding that in societies underpinned by racialized social hierarchies and with histories of race-based social stratification, including New Zealand, the labels ‘race’ and ‘ethnicity’ are not simply a matter of self-identification or cultural affiliation, but are also externally ascribed to individuals and groups as part of the racialization process [4], [5]. While internal and external processes of racial-ethnic identification are co-constitutive [4], Brodkin distinguishes between the concepts of “ethno-racial identity” and “ethno-racial assignment”:

Assignment is about popularly held classifications and their deployment by those with national power to make them matter economically, politically and socially to the individuals classified. We construct ethno-racial identities ourselves, but we do it within the context of ethno-racial assignment (p. 37, cited in [6]).

This external assignment of race/ethnicity may be based on interpretation of physical characteristics [6], [7], reflecting the purported biological underpinnings of racial categorizations, but may also draw on other features seen by society to be commonsense markers of race/ethnicity, such as name, language, style of dress [4], nationality, religion [6], as well as contextual factors and social stereotypes [7].

In racialized societies, the way in which individuals are categorized and assigned by others to racial/ethnic categories will influence their lived experiences [5]. As such, it has been proposed that socially-assigned race/ethnicity may have utility in examining the health effects of racism as it “…measures the *ad hoc* racial classification upon which racism operates” (p. 496, [5]). In this paper, racism is understood as a system that encompasses inequitable power relations, ideologies of race and accompanying processes of racialization, as well as discriminatory actions [8], [9]. The processes by which groups are racialized, and come to have meaning within specific social contexts, are key elements of racism. In racially-stratified societies, external ascription of race/ethnicity can influence exposure to racially discriminatory processes and practices at both structural and interpersonal levels. This, in turn, may manifest as health advantage for ‘dominant’ ethnic groups due to differential exposure to factors that are health-protective or health-damaging, as well as differences in access to and quality of health care [10]. In line with a ‘relational’ approach to race/ethnicity [11], [12], the terms ‘dominant’ and ‘minority’ are used here not in their numerical sense, but to reference unequal power relations and the hierarchical nature of the racial categorization of social groups [6], allowing for an approach to inequities in health that acknowledges accrued privilege rather than focusing simply on health disadvantage [13].

While there is substantial evidence linking racism to adverse health outcomes and ethnic inequalities [14], [15], there is limited interrogation of relationships between externally-imposed racial/ethnic categorizations and health outcomes [16], or examination of how racialization as ‘White’ or as belonging to the ‘dominant’ group may operate to promote health advantage [12], [17]. A 2008 study in the United States using data from the ‘Reactions to Race’ module of the Behavioral Risk Factor Surveillance System (BRFSS) found that, irrespective of one's self-identified race, being socially-assigned as White was associated with health advantage (as measured by self-rated health status) [5]. In contrast, analysis of BRFSS data for the states of Michigan and Wisconsin demonstrated that there was not a health advantage for individuals who self-identified as belonging to a minority group but were socially-assigned as White, or a health disadvantage among self-identified White participants who were socially-assigned to any minority group [18]. A recent study of the links between socially-assigned race, racial discrimination in healthcare settings and use of preventive healthcare services [19] demonstrated less exposure to healthcare discrimination among individuals who self-identified with a racial/ethnic minority group and were socially assigned as white, compared to participants who both self-identified and were socially-assigned as minority. Receipt of influenza and pneumococcal vaccination was higher among self-identified racial/ethnic minority participants who were socially-assigned as White, compared with those who were socially-assigned as a minority. Participation in cancer screening, however, was not significantly different between the different self-identified/socially-assigned groups [19].

This current study hypothesizes that in societies with contexts of colonization and settlement involving historical and contemporary racial categorization, such as New Zealand [20], [21], being socially-assigned as belonging to the dominant European ethnic grouping will be associated with health advantage. Secondly, it is proposed that this association will in part be due to lower exposure to racial discrimination at an individual level. In order to explore these hypotheses, we use data from the 2006/07 New Zealand Health Survey [22] to a) examine the associations between socially-assigned ethnicity, self-identified ethnicity, and health; and, b) consider the role of self-reported experience of racial discrimination in any relationships between socially-assigned ethnicity and health.

## Methods

Ethics approval for the current study (secondary analysis of 2006/07 New Zealand Health Survey data) was granted by the New Zealand Health and Disability Multi-Regions Committee (MEC/10/050/EXP).

Data for this study are from the adult sample of the 2006/07 New Zealand Health Survey (NZHS). The NZHS collected self-reported data on individuals' physical and mental health, health risk and protective factors, and health service use. Participants were sampled from the usually resident population aged 15 years and over living in permanent private dwellings [22]. The survey design used multi-stage, stratified, probability-proportional-to-size sampling, with an area-based sampling frame based on meshblocks (small geographical areas of approximately 100 residents per meshblock). Households were selected within each sampled meshblock, and a respondent selected within each sampled household (for further detail, see [22]).

The 2006/07 NZHS was interviewer-administered and included 12,488 adult respondents, with an overall weighted response rate of 67.9%. Weighted response rates by ethnic groups were 67.5% for Māori, 70.2% for Pacific peoples, 79.6% for Asian, 67.8% for European/Other ethnic groups [22].

### Key variables

#### Self-identified ethnicity

Self-identified ethnicity was collected for each participant using the 2006 New Zealand Population Census ethnicity question. This question asked ‘Which ethnic group or groups do you belong to?’ Individuals could choose from a list of eight response items or select ‘Other’ and provide a written response. Multiple responses were allowed, with participants self-identifying with more than one ethnic group counted in each of the broad ethnic categories they identified with. Responses were grouped into aggregate categories of Māori, Pacific, Asian, Other and European ethnic groups.

#### Socially-assigned ethnicity

This question asked: ‘Earlier you told me your ethnicity. Now I will ask you some questions about *reactions* to your ethnicity. Looking at Card 5.02, how do *other people* usually classify you in New Zealand?’ Participants were provided with the same response options as for the self-identified ethnicity question, and were able to report more than one socially-assigned ethnicity. As with self-identified ethnicity, responses were aggregated into broad categories of Māori, Pacific, Asian, Other, and European ethnic groups, with multiple responses counted in each ethnic grouping identified.

#### Ethnicity categories for analysis

To examine relationships between self-identified and socially-assigned ethnicity, we first grouped responses into mutually exclusive broad ethnic groupings, either single ethnicities or combinations of ethnicities (for those who identified with more than one ethnic group) across both self-identified and socially-assigned ethnicity. To look at the impact of being socially-assigned to the dominant ethnic grouping in New Zealand, data on both self-identified and socially-assigned ethnicity were further grouped into two broad categories: Dominant (Dom) or Minority (Min). These terms are used in this paper to reference power relations, rather than population size, and to reflect the focus of the study questions on dominant group advantage. The Dom category includes participants whose responses indicated they only identified with, or were assigned to, a European ethnic group or groups. All other responses were assigned to the Min group (including where individuals identified with, or reported being assigned to, both the dominant group and at least one minority group).

Four analytical categories were then created that classified people based on their responses to both the self-identified ethnicity and socially-assigned ethnicity questions: 1) people who reported they both self-identified and were socially-assigned only as dominant (SI Dom–SA Dom); 2) people who self-identified with a minority ethnic group, either alone or in combination with a European ethnic group, but reported they were only socially-assigned as dominant (SI Min–SA Dom); 3) people who self-identified only as dominant but reported being socially-assigned to a minority ethnic group, either alone or in combination with European ethnicity (SI Dom–SA Min); and, 4) people who both self-identified and were socially-assigned as belonging to a minority ethnic group, either alone or in combination with a European ethnic group (SI Min–SA Min). These analytical categories are similar to those used in other studies [18], [19]. For readability, these analytic categories will be referred to as SI Dom–SA Dom, SI Min–SA Dom, SI Dom–SA Min, and SI Min–SA Min in the text.

#### Health outcomes

Two health outcomes, self-rated health and psychological distress, were considered. Self-rated health was assessed using the first item of the SF-36, which asks: ‘In general, would you say that your health is: Excellent, Very good, Good, Fair, or Poor?’ This question has been validated and used widely in New Zealand and elsewhere to assess self-rated health [23], [24]. In line with the study's aim of exploring health advantage associated with socially-assigned ethnicity and the limited literature in this field [5], self-rated health was categorized into Excellent/Very good vs. Good/Fair/Poor. Psychological distress was measured with the Kessler 10-item (K10) scale, a measure that assesses symptoms of anxiety and depression in the last four weeks [25]. K10 was analyzed as a continuous outcome variable as these scores are known to be right skewed in the general population, rather than normally distributed. Group comparison of means are still valid, as the central limit theorem dictates that in a large sample the standard error (and by extension confidence intervals) will be a valid estimate of sampling variation processes, and hence for examining differences between groups [26]. These methods have been applied previously to analysis of K10 data from complex surveys in New Zealand [27].

#### Racial discrimination

Self-reported racial discrimination was assessed with five questions about individuals' experiences of ethnically-motivated a) verbal attack and/or b) physical attack, and unfair treatment on the basis of ethnicity c) by a health professional, d) at work or in obtaining work and/or e) in renting or buying housing. Exposure was measured as lifetime experience, i.e. ever experiencing racial discrimination. For descriptive analysis, racial discrimination variables are firstly presented for each item and then summarized as a 3 level factor (classifying whether people had reported no experiences, one experience, or two or more experiences across all four domains of racism). In multivariable analyses, racial discrimination was adjusted for using this more detailed three level factor.

### Covariates

Age was categorized into age bands (15–24, 25–34, 35–44, 45–54, 55–64, 65–74, 75+ years), and gender was assessed as male and female.

Educational qualification was classified as a dichotomous variable of reporting no secondary school (high school) qualification vs. any secondary school or post-secondary school qualification. The Economic Living Standards Index short form (ELSI-SF), which asks questions about restrictions in terms of material possessions and participation in social activities, and economic constraints, assessed living standards [28]. Scores were calculated and grouped into seven levels: severe hardship; significant hardship; some hardship; fairly comfortable living standards; comfortable living standards; good living standards; and, very good living standards. The New Zealand Index of Socioeconomic Deprivation for Individuals (NZiDep) was used to measure individual deprivation. NZiDep assigns respondents to five categories based on responses to eight questions: no deprivation characteristics (category 1); one deprivation characteristic (category 2); two deprivation characteristics (category 3); three or four deprivation characteristics (category 4); and, five or more deprivation characteristics (category 5) [29]. Area deprivation was measured with the New Zealand Index of Deprivation 2006 (NZDep2006), a measure of relative deprivation based on the deprivation score of the meshblock that the respondent lives in, from 1 being the least deprived to 10 the most deprived. The NZDep2006 score is calculated by combining responses to nine questions in the Population Census: access to a telephone; access to a car; qualifications; living space; home ownership; employment status; house-hold income; benefit receipt; and, living in a single-parent family [30].

### Analytical process

Data was analyzed in SAS 9.2 (SAS Institute, NC) using Survey analysis based procedures, with stratification and clustering elements of survey design taken into account. Data were weighted for probability of selection and non-response using survey weights to produce representative estimates for the New Zealand adult population and to calculate appropriate 95% confidence intervals.

Unweighted frequencies and weighted prevalence of demographic variables, self-rated health and experiences of racial discrimination were calculated for the four self-identified– socially-assigned analytical categories. Weighted means, medians and interquartile ranges were estimated for K10 scores for each self-identified–socially-assigned ethnic grouping.

Multivariable logistic regression analysis was undertaken to examine the relationship of the four self-identified–socially-assigned ethnic groupings to self-reported racial discrimination. Multiple regression models were used to test the likelihood of reporting good physical health (i.e. very good or excellent self-rated health) for each of the self-identified–socially-assigned ethnic groupings. Age, gender, education, living standards, individual deprivation, area deprivation, and racial discrimination were included sequentially. Age and gender were conceptualized as potential confounding variables, while the socioeconomic and racial discrimination measures were considered potential pathway variables. Modeling of K10 scores was conducted using Proc Surveyreg, using a linear model approach. Model covariates, and the sequential sets of covariates adjusted for at each stage of modeling, were the same as described for the self-rated health analysis above.

## Results

Among the 12,488 survey respondents, there was variable agreement between individuals' self-identified ethnicity and how they reported their socially-assigned ethnicity (at the level of broad ethnic groupings) (Table 1). Concordance between self-identified and socially-assigned ethnicity was high for people who self-identified as belonging to the dominant European-only ethnic grouping (97.6%) and the Asian-only ethnic grouping (92.7%). Levels of agreement were lower for Māori-only (79.9%), Pacific-only (70.7%), and Other-only (47.8%) self-identified groups. In general, disagreement between self-identified and socially-assigned ethnicity was higher where people self-identified with more than one broad ethnic grouping. For example, among those who self-identified as both Māori and European, 32.4% said they were usually socially-assigned as Māori only, 47.0% as European only, and 15.2% as both Māori and European.

**Table 1:** Cross-tabulation of self-identified ethnicity versus socially-assigned ethnicity^a^ (unweighted frequencies and percentages).

	Socially-assigned ethnicity										
Self-identified	E^b^ only	M only	M, E	P only	P, E	A only	A, E	O only	All else	*Missing*	Total n
**E only**	6708	47	51	-	-	6	8	7	6	*34*	6873
	(97.60%)	(0.68%)	(0.74%)	-	-	(0.09%)	(0.12%)	(0.10%)	(0.09%)	*(0.49%)*	
**M only**	113	1327	64	45	-	11	-	-	75	*19*	1660
	(6.81%)	(79.94%)	(3.86%)	(2.71%)	-	(0.66%)	-	-	(4.52%)	*(1.14%)*	
**M, E**	635	438	205	14	-	-	-	-	33	*15*	1351
	(47.00%)	(32.42%)	(15.17%)	(1.04%)	-	-	-	-	(2.44%)	*(1.11%)*	
**P only**	13	92	-	538	-	16	-	-	88	*8*	761
	(1.71%)	(12.09%)	-	(70.70%)	-	(2.10%)	-	-	(11.56%)	*(1.05%)*	
**P, E**	41	39	8	21	9	-	-	-	16	*-*	138
	(29.71%)	(28.26%)	(5.80%)	(15.22%)	(6.52%)	-		-	(11.59%)	*-*	
**A only**	17	6	-	6	-	1291	20	-	30	*16*	1392
	(1.22%)	(0.43%)	-	(0.43%)	-	(92.74%)	(1.44%)	-	(2.16%)	*(1.15%)*	
**A, E**	26	-	-	-	-	17	8	-	8	*-*	69
	(37.68%)	-	-	-	-	(24.64%)	(11.59%)	-	(11.59%)	*-*	
**O only**	20	-	-	-	-	-	-	33	-	*-*	69
	(28.99%)	-	-	-	-	-	-	(47.83%)	-	*-*	

^a^ This table includes 12,313 respondents, 98.6% of the total sample. The remaining respondents self-identified as belonging to other combinations of the ethnic groupings. This table summarizes agreement at the level of broad ethnic groupings (e.g. Asian) and does not measure disagreement that may exist within these broad groupings.

^b^ E = European, M = Māori, P = Pacific, A = Asian, O = Other.

Note: Cells with 5 or less respondents are suppressed in this table.

Table 2 shows the proportion of survey respondents in each of the four self-identified–socially-assigned analytical categories. The majority of people who self-identified only with the European ethnic grouping (SI Dom) reported they were usually socially-assigned as only being European (SA Dom) by others (97.6%). The proportion of people self-identifying only as European (SI Dom) but reporting they were socially-assigned to a non-dominant ethnic group, or to both the dominant and one or more minority ethnic groups (SA Min), was relatively small (2.4%). Among respondents in the self-identified minority group (SI Min), 84.1% said that they were usually socially-assigned to a minority ethnic group as their only, or one of their, socially-assigned ethnic groups (SA Min). However, 16% of people who self-identified with a minority ethnic group (as their only self-identified ethnic group or one of their self-identified ethnicities) reported being socially-assigned to the dominant ethnic group (SA Dom).

**Table 2:** Cross-tabulation of four self-identified–socially-assigned analytical categories (unweighted frequencies and percentages).

	Socially-assigned dominant only (SA Dom)	Socially-assigned minority ever (SA Min)
	n (%)	n (%)
**Self-identified dominant only (SI Dom)**	6708 (97.6)	165 (2.4)
**Self-identified minority ever (SI Min)**	893 (15.9)	4722 (84.1)

Individuals who self-identified with both dominant and minority ethnic groups were included in the SI Min analytical category. Among this group, 29% (n = 1,646) identified as belonging to both the dominant European group and one or more minority group, while 71% (n = 3,969) identified only with a minority group or groups. Of individuals who self-identified with both the dominant European group and one or more minority ethnic groups, 44% reported they were usually socially-assigned as only belonging to the dominant European grouping, 40% reported that they were usually classified as only belonging to a minority ethnic group, and 16% reported being socially-assigned to both. The majority of people in the analytical category SI Min–SA Dom self-identified as belonging to both the dominant and one or more minority group (727 of 893 people) (data not shown in table).

Descriptive analyses suggest some differences in socio-demographic characteristics between the four self-identified–socially-assigned ethnicity analytical categories (Table 3). The group SI Dom–SA Dom tended to have an older age distribution and greater socioeconomic advantage than the other three groups (Table 4). The group SI Min–SA Min tended to report the highest prevalence of exposure to racial discrimination and was more likely than the SI Dom–SA Dom and SI Min–SA Dom groups to report multiple (2+) experiences of racial discrimination (Table 4). These patterns were also reflected in logistic regression analyses of experience of racial discrimination (Table 5), which showed that after adjusting for age, sex and socioeconomic position, SI Min–SA Min had 2.5 times higher odds of ever experiencing racial discrimination compared with SI Dom–SA Dom. The groups SI Min-SA Dom and SI Dom-SA Min were intermediate.

**Table 3:** Socio-demographic characteristics of the four self-identified–socially-assigned analytical categories (weighted percentages).

Characteristic	SI Dom–SA Dom	SI Min–SA Dom	SI Dom–SA Min	SI Min–SA Min
	n (%)	n (%)	n (%)	n (%)
**Gender**				
Female	3778 (51.7)	557 (53.6)	97 (54.8)	2783 (52.4)
Male	2930 (48.3)	336 (46.4)	68 (45.2)	1939 (47.6)
**Age group**				
15–24 years	531 (14.1)	192 (28.2)	22 (18.6)	918 (27.4)
25–34 years	785 (14.3)	196 (22.4)	28 (17.6)	1071 (21.3)
35–44 years	1196 (18.9)	188 (19.5)	40 (26.0)	1153 (21.2)
45–54 years	1134 (18.6)	132 (14.8)	24 (15.6)	789 (16.0)
55–64 years	1181 (15.3)	93 (8.1)	24 (12.1)	431 (8.1)
65–74 years	968 (10.2)	60 (4.6)	12 (4.6)	264 (4.4)
75+ years	913 (8.5)	32 (2.3)	15 (5.6)	96 (1.5)
**Education**				
No secondary	2022 (25.8)	298 (27.7)	61 (32.4)	1626 (29.2)
Secondary education	4675 (74.2)	595 (72.3)	104 (67.6)	3082 (70.8)
**ELSI**				
Severe hardship	76 (0.9)	25 (1.7)	9 (3.6)	185 (3.0)
Significant hardship	113 (1.2)	37 (4.6)	8 (4.8)	253 (4.3)
Some hardship	261 (3.5)	64 (7.1)	16 (10.0)	387 (7.2)
Fairly comfortable	499 (7.6)	100 (8.7)	13 (7.3)	662 (14.8)
Comfortable	1224 (18.7)	206 (23.4)	34 (23.0)	1098 (24.4)
Good	3043 (45.1)	322 (39.2)	63 (42.1)	1648 (37.6)
Very good	1457 (22.9)	123 (15.2)	15 (9.3)	380 (8.6)
**Area deprivation**				
Decile 1 (least deprived)	723 (12.8)	64 (7.7)	12 (9.7)	164 (3.6)
Decile 2	733 (12.9)	64 (8.4)	15 (9.8)	236 (5.8)
Decile 3	667 (9.9)	70 (8.0)	10 (5.8)	218 (4.7)
Decile 4	744 (11.8)	90 (11.2)	17 (12.5)	306 (7.9)
Decile 5	783 (11.3)	76 (8.2)	14 (10.3)	352 (8.3)
Decile 6	748 (10.7)	101 (9.4)	19 (11.8)	408 (8.6)
Decile 7	707 (10.3)	112 (13.4)	18 (12.5)	539 (11.3)
Decile 8	646 (8.5)	112 (12.6)	15 (8.6)	582 (13.6)
Decile 9	521 (6.9)	84 (10.1)	23 (10.1)	680 (13.1)
Decile 10 (most deprived)	436 (5.0)	120 (10.9)	22 (8.8)	1237 (23.2)
**Individual deprivation**				
Category 1 (least deprived)	4851 (72.0)	478 (54.5)	83 (51.5)	2236 (50.4)
Category 2	993 (16.0)	182 (22.9)	29 (18.8)	989 (22.5)
Category 3	383 (5.9)	90 (9.3)	21 (15.2)	540 (10.5)
Category 4	323 (4.2)	94 (9.3)	14 (6.9)	614 (11.6)
Category 5 (most deprived)	151 (1.9)	49 (4.0)	18 (7.6)	333 (5.0)

**Table 4:** Prevalence of racial discrimination exposures and health outcomes by the four self-identified–socially-assigned ethnicity categories^a^.

Characteristic	SI Dom–SA Dom	SI Min–SA Dom	SI Dom–SA Min	SI Min–SA Min
	n	n	n	n
	% (95% CI)	% (95% CI)	% (95% CI)	% (95% CI)
**Racial discrimination**				
Verbal assault (ever)	702	173	27	1106
	10.6% (9.7, 11.5)	18.2% (14.9, 21.6)	17.3% (10.3, 24.3)	22.5%, (20.8, 24.1)
Physical assault (ever)	163	29	10	232
	2.8% (2.3, 3.4)	3.0% (1.5, 4.4)	7.5% (2.2, 12.8)	4% (3.4, 4.6)
Discrimination in health (ever)	76	35	8	260
	1.0% (0.8, 1.3)	3.1% (1.9, 4.4)	3.7% (0.7, 6.6)	5.1% (4.2, 5.9)
Discrimination in work (ever)	113	33	9	414
	1.8% (1.4, 2.2)	4.6% (2.5, 6.7)	5.5% (1.5, 9.4)	8.7% (7.7, 9.7)
Discrimination in housing (ever)	30	25	8	376
	0.4% (0.2, 0.6)	2.0% (1.0, 3.0)	3.2% (0.0, 6.5)	6.4% (5.6, 7.2)
No experiences	5835	677	122	3157
	86.8% (85.7, 87.8)	76.8% (72.9, 80.7)	75.6% (67.8, 83.4)	68.7% (66.9, 70.5)
One experience	676	151	25	967
	10.3% (9.4, 11.2)	17.7% (14.3, 21.0)	13.1% (7.4, 18.8)	20.5% (18.9, 22.0)
Two or more	190	59	16	572
	3.0% (2.5, 3.5)	5.6% (3.7, 7.4)	11.3% (5.1, 17.6)	10.8% (9.7, 11.9)
**Self-rated health**				
Excellent self-rated health	1306	137	29	710
	20.8% (19.6, 22.0)	14.0% (11.0, 17.0)	19.4% (12.2, 26.7)	16.3% (14.9, 17.7)
Very good self-rated health	2806	354	53	1702
	42.7% (41.3, 44.1)	43.5% (38.7, 48.3)	32.2% (23.6, 40.8)	36.1% (34.2, 38.0)
Good self-rated health	1873	301	54	1674
	27.0% (25.7, 28.3)	31.8% (27.3, 36.3)	32.0% (23.8, 40.2)	35.0% (33.2, 36.9)
Fair self-rated health	604	79	22	515
	7.8% (7.1, 8.5)	8.2% (5.8, 10.5)	13.9% (7.5, 20.4)	10.3% (9.2, 11.4)
Poor self-rated health	118	22	7	121
	1.7% (1.3, 2.0)	2.5% (1.2, 3.8)	2.4% (0.2, 4.7)	2.3% (1.8, 2.7)
**Kessler 10 (psychological distress)**				
Mean (95% CI)	3.17	5.08	4.31	4.27
	(3.04, 3.30)	(4.07, 6.09)	(3.81, 4.80)	(4.05, 4.49)
Median (Interquartile range)	1.3	2.1	2.6	1.8
	(0.0, 3.8)	(0.2, 5.1)	(0.0, 6.5)	(0.0, 5.4)

^a^ Unweighted frequencies (n); weighted value for percentages, means, and medians.

**Table 5:** Odds of reporting ever experiencing racism for the self-identified–socially-assigned ethnicity categories.

	Self-identified–socially-assigned ethnicity categories			
	SI Dom–SA Dom	SI Min–SA Dom	SI Dom-SA Min	SI Min–SA Min
		OR (95% CI)	OR (95% CI)	OR (95% CI)
**Unadjusted model**	Reference	1.98 (1.57, 2.51)	2.11 (1.38, 3.24)	2.98 (2.64, 3.37)
**Adjusted for age, sex**	Reference	1.82 (1.43, 2.32)	2.01 (1.31, 3.07)	2.71 (2.39, 3.08)
**Adjusted for potential pathway variables**				
+ qualification	Reference	1.84 (1.44, 2.34)	2.04 (1.33, 3.11)	2.75 (2.42, 3.13)
+ ELSI, NZiDep, NZDep06	Reference	1.68 (1.32, 2.14)	1.88 (1.23, 2.87)	2.49 (2.18, 2.85)

In terms of health outcomes, the group SI Dom–SA Dom was significantly more likely to report excellent/very good health than the SI Min–SA Min group (Table 4, unadjusted estimates in Table 6). The SI Dom–SA Dom grouping had the lowest mean K10 scores, with the other groups all having significantly higher mean K10 scores compared with SI Dom–SA Dom (Table 4; unadjusted estimates in Table 7).

**Table 6:** Odds of reporting excellent/very good health (compared to good/fair/poor health) for the self-identified–socially-assigned ethnicity categories.

	Self-identified–socially-assigned ethnicity categories			
	SI Dom–SA Dom	SI Min–SA Dom	SI Dom–SA Min	SI Min–SA Min
		OR (95% CI)	OR (95% CI)	OR (95% CI)
**Unadjusted model**	Reference	0.78 (0.63–0.96)	0.61 (0.43–0.88)	0.63 (0.58–0.70)
**Adjusted for age, sex**	Reference	0.70 (0.56–0.86)	0.57 (0.39–0.81)	0.56 (0.51–0.62)
**Adjusted for potential pathway variables**				
+ qualification	Reference	0.72 (0.58–0.88)	0.59 (0.41–0.85)	0.58 (0.53–0.65)
+ ELSI, NZiDep, NZDep06	Reference	0.82 (0.66–1.02)	0.77 (0.53–1.11)	0.76 (0.67–0.85)
+ Racism	Reference	0.85 (0.68–1.05)	0.80 (0.55–1.15)	0.79 (0.70–0.89)

**Table 7:** Differences in mean K10 scores for the self-identified–socially-assigned ethnicity categories.

	Self-identified–socially-assigned ethnicity categories			
	SI Dom–SA Dom	SI Min–SA Dom	SI Dom–SA Min	SI Min–SA Min
		Mean Difference (95% CI)	Mean Difference (95% CI)	Mean Difference (95% CI)
Unadjusted model	Reference	1.16 (0.64, 1.68)	1.84 (0.77, 2.90)	1.04 (0.79, 1.29)
Adjusted for age, sex	Reference	0.94 (0.42, 1.46)	1.73 (0.68, 2.78)	0.84 (0.59, 1.10)
**Adjusted for potential pathway variables**				
+ qualification	Reference	0.88 (0.35, 1.40)	1.63 (0.61, 2.65)	0.76 (0.50, 1.02)
+ ELSI, NZiDep, NZDep06	Reference	0.36 (−0.10, 0.82)	0.77 (−0.22, 1.77)	−0.06 (−0.31, 0.20)
+ Racism	Reference	0.27 (−0.18, 0.71)	0.64 (−0.36, 1.64)	−0.27 (−0.52, −0.02)

In adjusted models, individuals in the SI Dom–SA Dom group had a significant health advantage compared with the other groups after adjusting for age and sex (Table 6). SI Dom–SA Min (OR 0.57, 95% CI 0.39–0.81) and SI Min–SA Min (OR 0.56, 95% CI 0.51–0.62) had the lowest odds of reporting excellent/very good health, and similar point estimates. The group SI Min–SA Dom (OR 0.70, 95% CI 0.56–0.86) had poorer self-rated health than SI Dom–SA Dom but this difference was less pronounced than for the two socially-assigned minority groups (SI Dom–SA Min and SI Min–SA Min).

After controlling for socioeconomic position and individual exposure to racial discrimination, the associations between ethnicity and self-rated health were attenuated, although point estimates remained below 1 (Table 6). The odds of reporting excellent/very good self-rated health remained significantly lower for the SI Min–SA Min group compared with the SI Dom–SA Dom group (OR 0.79, 95% CI 0.70–0.89), although the point estimate was similar to SI Min–SA Dom and SI Dom–SA Min.

In analysis of the K10 outcomes, compared with the SI Dom–SA Dom group, all other groups had significantly higher mean levels of psychological distress after adjusting for age and sex, with the highest difference found in the SI Dom–SA Min grouping, followed by SI Min–SA Dom and SI Min–SA Min (Table 7). Controlling for socioeconomic position and racial discrimination attenuated these differences. The SI Min–SA Min group had significantly lower psychological distress scores than the SI Dom–SA Dom group after adjusting for all variables.

## Discussion

The current study demonstrates a complex relationship between self-identified ethnicity, socially-assigned ethnicity, and measures of health and racial discrimination. Analysis identified disagreement between reporting of self-identified and socially-assigned ethnicity, with levels of discordance differing by broad ethnic grouping. For individuals who self-identified with more than one ethnic group, some of this discordance may have been related to the wording of the socially-assigned ethnicity question, which primed participants to give a single ethnicity as a response. However, this finding is consistent with other research showing differing levels of agreement between self-identified and socially-assigned race/ethnicity by self-identified racial/ethnic group [5], [18], [19], [31]. A Canadian study, for example, found that concordance was highest between self-identified and self-reported socially-assigned race for White and Asian groups, with lower agreement among Black and South Asian individuals [31]. In the United States, agreement has been shown to be high for Black and White (non-Hispanic White) groups, and lower for Hispanic individuals [19].

Respondents who both self-identified and reported being socially-assigned as belonging to the dominant European grouping (SI Dom–SA Dom) had the highest odds of reporting optimal self-rated health. The other three groupings all had significantly lower self-rated general health, with this difference remaining significant after taking socioeconomic measures and racial discrimination into account for those who both self-identified and were socially-assigned as ever belonging to a minority ethnic grouping (SI Min–SA Min). In terms of general health status, there appeared to be some health advantage associated with being always socially-assigned as European among respondents who self-identified with at least one minority ethnic group (SI Min–SA Dom), with point estimates higher for this group than for the two socially-assigned minority groups, although this was not statistically significant. The findings also suggest that (ever) self-identifying or being socially-assigned to a minority group (or groups) is linked to lower odds of reporting excellent or very good health, in part due to increased socioeconomic disadvantage and exposure to racial discrimination.

As with general health, the group SI Dom–SA Dom had the lowest levels of psychological distress adjusted for age and sex. Differences with other groups were attenuated, however, after controlling for socioeconomic status and racial discrimination. Following adjustment for all variables, the mean K10 score for the SI Min–SA Min became significantly lower than the SI Dom–SA Dom group, suggesting that exposure to racial discrimination and socioeconomic disadvantage are important drivers of inequalities in psychological distress between these groups. The group that self-identified as always dominant but reported that they were (ever) socially-assigned to a minority ethnicity had the highest age, sex adjusted levels of psychological distress, although the sample size was small and the difference was not statistically significant. However, this is consistent with a Canadian study that found respondents who self-identified as White but reported being assigned by others to another ethnic group had a higher likelihood of reporting lower mental health than respondents who both self-identified and reported being assigned by others as White [31]. It has been suggested that this ‘mismatch’ acts as a stressor because it challenges the typically normalized, ‘non-racial’ identity that many White people have, positing that “…most White Canadians typically do not see or experience ‘race’ as other Canadians do; their racial identities are experienced as normal, taken-for-granted, straightforward, and obvious” (p. 1160, [31]). It may also be capturing something about the different experiences that individuals with discordant self-identified and socially-assigned race/ethnicity may have compared with those for whom there is concordance [32]. The study by Veenstra [31] found both physical and mental health effects of disagreement between self-identified and socially-assigned ethnicity. Other studies have also found a complicated relationship between race/ethnicity, discrimination and psychological distress [16].

In analysis from the United States, respondents who self-identified and were socially-assigned as Asian had the highest levels of health status, followed by those self-identifying and socially-assigned as White [5]. In contrast to our study, Jones et al. [5] also found being socially-assigned as White was associated with an increased likelihood of reporting excellent or very good health regardless of how individuals self-identified. Similarly, recent analysis of the relationships between socially-assigned race/ethnicity and preventive healthcare use found that being socially-assigned as White was associated with increased receipt of vaccinations among individuals who self-identified with a racial/ethnic minority group [19], although there was no significant association demonstrated in this study between socially-assigned as White and receipt of cancer screening in adjusted analyses. Ridings, Rafferty, and Weir [18] did not demonstrate either a physical or mental health advantage for respondents from minority groups who were socially-assigned as White. They suggest that the observed potential advantage associated with being socially-assigned as White among minority respondents may be restricted in their study because of the structural contexts within which their sample resided (in Detroit, MI and Milwaukee, WI), and the impact of segregation and historical systemic disadvantage on minority group members irrespective of their social-assignment. They note, “…privilege associated with Whiteness is not only found in interpersonal interactions but also in the community-level aggregation of privilege, which is expressed in larger social constructs such as city planning, environmental policy, and transportation” (p. 2, [18]). This broader structural context is likely to also be a factor in our study, and is represented to some degree by the differential distribution of socioeconomic measures across the self-identified–socially-assigned ethnic groupings.

This study has several limitations that need to be considered. Firstly, the question used to measure socially-assigned race/ethnicity in the NZHS is respondents' perceptions of how others usually view their ethnic group, rather than a direct measure of social-assignation, such as observer-reported race/ethnicity [32]. In this sense it captures information on ‘reflected race’ (the race an individual thinks they are usually assigned to) [33]. While there is some evidence of discordance between self-identified and observer-assigned race/ethnicity [32], [33], the extent to which an individual's appraisal of how they are usually socially-assigned captures actual practices of social ascription is unclear, particularly in New Zealand. In cognitive testing of questions on socially-assigned ethnicity and socially-assigned race with a sample of English- and Spanish-speaking adults in the United States, most study participants understood the question to be about how they felt other people perceived them [34]. However, the authors also found through discussing with participants how they had come to their response that participants were in fact reflecting their self-defined identity [34]. Yet the discordance between self-identified and ‘reflected’ race/ethnicity data found in other studies [5], [18], [19] suggests that there is a distinction between the self-identified and socially-assigned constructs, at least for some racial/ethnic groups. Additionally, the question used to assess socially-assigned ethnicity in the current study asked broadly about how people felt they were usually classified, without providing any specific context, such as whether or not the ascription was by strangers. There is some evidence that context plays a role in how observers assign racial identity to others [7].

Interpreting the socially-assigned ethnicity data in this study was particularly complex as some survey participants gave multiple responses. Where this occurred, it was unclear whether these individuals were reporting they were usually socially-assigned as being ‘multi-ethnic’ or belonging to more than one group, or that they were sometimes socially-assigned to one group and other times to another group. Furthermore, due to the number of people within specific ethnic groups and the potential range of different combinations of multiple ethnicities, we were only able to examine agreement between self-identified and socially-assigned ethnicity at the level of aggregate ethnic groupings, such as European and Asian (see Table 1). This approach potentially masked disagreement within these broader groupings and is, therefore, likely to underestimate true levels of discordance between self-identified and socially-assigned ethnicity. It also meant it was not possible to examine health impacts potentially associated with the assignment of individuals to a single ethnic group among those who self-identified with multiple ethnic groups.

Further, to examine advantage among individuals who were always socially-assigned to the dominant group, individuals who reported either being self-identified or socially-assigned as belonging to both a dominant and a minority group were assigned to the (ever) minority group for the purposes of analysis. In comparing all respondents who self-identified or were socially-assigned to a minority ethnic grouping to the dominant group, we were not able to explore the differences in experiences, exposures and outcomes between the various ethnic groups or assess whether the relationships differed by ethnic group (although analysis focusing specifically on Māori is being undertaken and will be reported elsewhere).

The measures of racial discrimination available in the NZHS assess only some forms of ethnically-motivated discrimination experienced and reported by individuals, and do not capture the full range of discrimination experiences, including those which respondents may not be able to articulate [35]. As they are survey-derived, they are also cross-sectional and based on individuals' recall and are subject, therefore, to the usual limitations of cross-sectional, self-report data.

Strengths of the study include that it is the first to report on socially-assigned ethnicity and health in New Zealand, and draws on data from a large, nationally-representative sample. In addition, the study examined the impact of racial discrimination on relationships between socially-assigned ethnicity and health. As predicted, and consistent with patterns of racial discrimination by self-identified ethnic group [36], [37], there were differences in reported racial discrimination by socially-assigned ethnicity. Being socially-assigned to and/or self-identifying with the dominant European grouping was associated with reduced exposure to racial discrimination in New Zealand, a known health determinant. This is consistent with a recent study in the United States, which found being socially-assigned as White to be associated with lower reported experience of healthcare discrimination [19].

In line with the dialectical nature of the formation of identity [4], [38], self-identified and socially-assigned race/ethnicity are inter-related and necessarily inform each other. However, they also capture different aspects of living in a racialized society [38], as they reflect differing processes of racialization [33]. There is increasing support for viewing racial/ethnic identities as what Veenstra terms “a multifaceted or multidimensional suite of interlinked identities” (p. 1160, [31]). In delineating these facets of racialized identities, Roth [33] distinguishes between ‘internal race’ (the identity someone holds at an individual level) and ‘expressed race’ (the racial identity that an individual expresses to others, such as in filling out official questions). In terms of externally applied racial labels, Roth [33] outlines concepts of ‘reflected race’ (the race an individual thinks they are usually assigned to, corresponding with the current study's measure of socially-assigned race), and ‘observed race’ (the racial identity assigned by an observer). In this approach, there is not one ‘correct’ measure of racial/ethnic identity, but rather various alternatives [33], [39], which all may be associated with different experiences of racial discrimination [31].

The current study supports an approach to research on racial/ethnic health inequities that recognizes the variant ways in which individuals and groups are racialized and the meaning that this has for their everyday experiences and realities [33], including their exposures to health-damaging or health-protective factors and resultant health impacts [3]. In examining relationships between self-identified and socially-assigned ethnicity, this study facilitates an understanding of racial/ethnic identity as socially-constructed and contingent, encouraging increased interrogation of the pathways by which privilege accrues to ‘dominant’ racial/ethnic groups in racially-stratified societies [13]. The health advantage for people who both always self-identified and were socially-assigned as European appeared to be related to their relatively higher socioeconomic position and lower exposure to racial discrimination, demonstrating privileged access to the determinants of good health for the ‘dominant’ ethnic grouping in New Zealand.

In conclusion, in racialized societies such as New Zealand, both self-identified and socially-assigned ethnicity appear to be important for physical and mental health outcomes. Self-identified ethnicity may be capturing important dimensions of structural racism that are manifest in racially-stratified exposures and opportunities [18], as well as the inter-generational and cumulative impacts of racism. As self-identified ethnicity is the most routinely collected ethnicity variable in New Zealand, it is an important source of data for measuring and monitoring population-level health status and inequalities between ethnic groups. However, socially-assigned race/ethnicity is also useful in measuring exposure to racial discrimination [5], [33] and provides another tool for assessing the health impacts of social processes of racialization. As Daniels & Schulz note, understanding ‘race’ within its social context is important in research as “the failure to explicitly conceptualize race as a set of social relations leaves descriptions of racial differences in biological or behavioral factors associated with differential health outcomes open to interpretations as produced through biological, genetic, or culturally patterned lifestyle differences” (p. 100, [13]). Socially-assigned ethnicity supports explanations for ethnic health inequalities that recognize the fundamental role of racialized social relations and experiences, in contrast to deficit accounts that seek to locate the ‘problem’ in individuals or communities themselves. It is important, then, to carefully consider the strengths and limitations of varying approaches to categorizing racial/ethnic identities in studies of inequalities [39] and in advancing our understanding of explanatory pathways.

This study is consistent with the broader evidence demonstrating the deleterious impacts of racism on health and ethnic inequalities. Racism affects health and ethnic inequalities by determining access to social goods and resources in systematic, institutionalized ways, through the harm, trauma and chronic stress caused by experiences of racial discrimination, and through the very processes and outcomes of racialization at societal levels. Given the relational, socially-constructed nature of race/ethnicity, the association between socially-assigned race/ethnicity and social outcomes will persist as long as racism persists, providing further imperative to the need to address racism as a public health issue in a concerted and committed way.
